# Effects of fresh frozen plasma, Ringer’s acetate and albumin on plasma volume and on circulating glycocalyx components following haemorrhagic shock in rats

**DOI:** 10.1186/s40635-016-0080-7

**Published:** 2016-03-03

**Authors:** Axel Nelson, Svajunas Statkevicius, Ulf Schött, Pär I. Johansson, Peter Bentzer

**Affiliations:** Department of Clinical Sciences, Section of Anaesthesiology and Intensive Care, Lund University and Skane University Hospital, Lund, Sweden; Section for Transfusion Medicine, Capital Region Blood Bank, Rigshospitalet, Copenhagen, Denmark; Department of Surgery, University of Texas Health Medical School, Houston, TX USA; Department of Anesthesia and Intensive Care, Helsingborg Hospital, Helsingborg and Lund University, Lund, SE-251 87 Sweden

**Keywords:** Resuscitation, Haemorrhage, Fresh frozen plasma, Heparan sulphate, Syndecan-1

## Abstract

**Background:**

Early use of fresh frozen plasma (FFP) in haemorrhagic shock is associated with improved outcome. This effect may partly be due to protection of the endothelial glycocalyx and/or secondary to a superior efficacy of FFP as a plasma volume expander compared to crystalloids. The objective of the present study was to investigate if protection of the glycocalyx by FFP can be demonstrated when potential differences in plasma volume (PV) following resuscitation are accounted for.

**Methods:**

Rats were subjected to a volume-controlled haemorrhage (30 ml/kg). At 2.5 h after haemorrhage, animals were randomized to resuscitation with FFP (37.5 ml/kg), albumin (30 ml/kg) or Ringer’s acetate (RA) (135 ml/kg, 4.5 times the bleed volume). PV was measured 2 h after completion of resuscitation using ^125^I-albumin and effects on endothelial glycocalyx were evaluated by measuring circulating heparan sulphate and syndecan-1. Hemodynamic effects of resuscitation were evaluated by measuring lactate and mean arterial pressure (MAP).

**Results:**

Resuscitation with FFP or albumin resulted in plasma volume expansion equalling the blood loss (to 55 ± 5 ml/kg and 54 ± 4 ml/kg (mean ± S.D.), respectively), whereas plasma volume expansion in RA group was lower (to 42 ± 7 ml/kg). Plasma concentration of heparan sulphate was lower in the FFP and albumin groups than in the RA group at 2 h after resuscitation. After correcting for differences in plasma volume, no significant difference in circulating amount of heparan sulphate was detected between the FFP and albumin groups (2879 ± 1075 μg/kg and 3318 ± 1814 μg/kg, respectively, *P* = 0.4) and the RA group (3731 ± 777 μg/kg). No differences between the groups in plasma concentration or amount of circulating syndecan-1 were detected after resuscitation. After resuscitation, MAP was higher in the FFP and albumin groups than in the RA group. Lactate did not differ between the FFP and RA groups after resuscitation.

**Conclusions:**

Improved outcome in trauma by FFP could in part be explained by better plasma volume expansion compared to crystalloids. The decrease in plasma concentration of markers of glycocalyx degradation after resuscitation with FFP are largely secondary to differences in plasma volume and may not accurately reflect effects of FFP on the glycocalyx.

## Background

Early use of fresh frozen plasma (FFP) in the resuscitation of trauma-induced haemorrhagic shock has been associated with improved outcome, but the underlying mechanisms are not fully understood [1]. Partly, the better outcome may be explained by improved coagulation [2], but other mechanisms are likely to contribute.

Endothelial cells are covered by a layer of membrane bound molecules including proteoglycans containing high concentrations of heparan sulphate such as syndecans and glypicans and loosely bound plasma proteins, collectively called the glycocalyx [3, 4]. It has been suggested that an intact glycocalyx is of importance for maintenance of vascular homeostasis and that shedding of the glycocalyx may induce increases in permeability, activation of coagulation system and leucocyte adherence [3–5]. Recent data shows that increased plasma levels of the proteoglycan syndecan-1 at arrival to hospital correlate with a poor outcome in trauma patients with similar injury severity scores [6] and suggest that degradation of the glycocalyx is an early consequence of severe trauma. Several experimental studies have shown that resuscitation with FFP may decrease both plasma concentration of syndecan-1 and attenuate decreases in thickness of the endothelial glycocalyx indicating that the beneficial effects of FFP in haemorrhagic shock in part could be explained by effects on the glycocalyx [7, 8]. In an attempt to separate direct effects of FFP on the glycocalyx from effects mediated via improved cardiac output, these studies compared resuscitation with FFP with crystalloids at a volume ratio of about 1:3 to obtain comparable plasma volume expansion. However, plasma volumes were not measured and may have been higher in animals resuscitated with FFP. If so, this is a potential mechanism by which FFP could mitigate shedding of the glycocalyx. FFP also contains high concentrations of albumin, which is a potential modulator of the endothelial glycocalyx and is possible that FFP acts by replacing albumin lost following haemorrhage [9–12].

Based on these considerations, the present study was designed to investigate if the previously observed protection of glycocalyx by FFP compared to crystalloids in haemorrhagic shock can be demonstrated when potential differences in plasma volume following resuscitation are accounted for. We also wanted to investigate if albumin could confer the same protective effects on the glycocalyx as FFP. For this purpose, rats were subjected to a volume-controlled haemorrhage followed by resuscitation with FFP, 5 % albumin or Ringer’s acetate. Dose of respective resuscitation fluid was chosen with the objective to achieve similar plasma volumes in all groups after resuscitation. Effect of resuscitation on plasma volume was measured using radiolabeled albumin, and effects on endothelial glycocalyx were evaluated by measuring plasma concentrations of heparan sulphate and syndecan-1. Potential effects of the different resuscitation fluids on permeability were evaluated by measuring transcapillary escape rate of albumin. The adequacy of cardiac output following resuscitation was evaluated by measurement of lactate and central venous saturation.

## Methods

### Anaesthesia and surgical preparation

The study was approved by the Ethics Committee for Animal research at Lund University, Sweden (M103-09), and the animals were treated in accordance with the National Institutes of Health for the Care and Use for Laboratory animals. Seventy-five adult male Sprague-Dawley rats (Scanbur BK, Sollentuna, Sweden) weighing 355 ± 17 g (mean ± SD) were used. The objective was to have 9–10 animals to complete the experimental protocol in the treatment groups. Substitutes were used for animals that died prior to completion of the protocol. All measurements except blood gas analysis were performed by an investigator blinded to the treatment status of the animals.

**Fig. 1:**
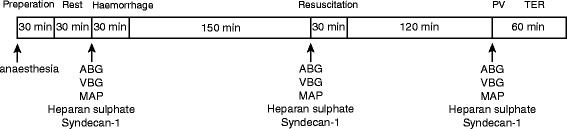
Overview of the experimental protocol. *ABG* arterial blood gas, *MAP* mean arterial pressure, *PV* plasma volume measurement, *TER* transcapillary escape rate of albumin, *VBG* venous blood gas

An overview of the experimental protocol is presented in Fig. 1. Anaesthesia was induced with 4 % isoflurane (Schering-Plough Animal Health, Ballerup, Denmark) in a closed container, and thereafter, the anaesthesia was maintained with 1.6–1.8 % isoflurane in air delivered via a mask until a tracheostomy and endotracheal intubation was performed and isoflurane was thereafter lowered to 1.1–1.3 %. Animals were mechanically ventilated (Ugo Basile Animal Ventilators, Comerio, Italy) to an end-tidal CO_2_ concentration of between 4.5 and 6.5 kPa using a volume-controlled mode and a positive end-expiratory pressure of 3 cm H_2_O. Body temperature, measured rectally, was maintained at 37 °C with a heating pad throughout the experiment. The left femoral artery was cannulated for measurement of arterial pressure, blood sampling and as a port for bleeding procedure. The left femoral vein was cannulated for administration of resuscitation fluids. The right internal jugular vein was cannulated for measurement of central venous blood gases and administration of ^125^I-albumin. After a 30-min equilibration period, mean arterial pressure was recorded and arterial and central venous blood gas, electrolytes, lactate and haematocrit were collected (I-stat, Hewlett Packard, Böblingen, Germany). Plasma samples were collected in heparinized vials and stored at −80 °C until analysis.

### Haemorrhage and resuscitation

The animals underwent controlled haemorrhage of 25 ml/kg during 15 min followed by equilibration for 10 min and then bled another 5 ml/kg during 10 min or a sham procedure. The volume of bleeding corresponds to about 48 % of total blood volume in the rat [13]. After 150 min, blood sample collection was performed. To ensure that the animals were similarly and severely affected by the haemorrhage, we excluded animals that had a lactate <3.5 mmol/l at this time point. The animals were then randomized to resuscitation with Ringer’s acetate (RA, Fresenius Kabi, Uppsala, Sweden; osmolality: 278 mOsm/kg) in a volume of 4.5 times the bleed volume or 5 % albumin (ALB, CSL Behring, Marburg, Germany; osmolality: 269 mOsm/kg) in a volume equal to the bleed volume or fresh frozen plasma (FFP, produced in house as seen below, osmolality: 317 mOsm/kg) in a volume of 1.25 times the bleed volume. The FFP preparation has an estimated albumin and protein content of 26 and 57 g/l, respectively [14]. The resuscitation fluids were pre-warmed to 37 °C and were administrated during 30 min. The volume of fluid in the RA group was chosen with the objective to achieve equal plasma volume expansion as in the ALB and FFP groups based on previous results in a similar model of haemorrhage [15]. The volume of FFP was expected to give equal plasma volume expansion as in the RA and ALB groups based on previously published data in humans and on pilot studies in rats [16]. Sham animals were neither bled nor resuscitated but in every other way treated exactly as above. Osmolality was measured with the freeze-point method (Micro-Osmometer Model 210; Fiske Associates, Norwood, Massachusetts).

### Preparation of fresh frozen plasma

Following induction of anaesthesia as described above, FFP was made by collecting about 10 ml of blood in 3–5 min from the cannulated femoral artery in donor rats. Blood was thereafter mixed with a citrate-phosphate-dextrose (CPD) solution (Compflow, Fresenius Kabi, Oberursel, Germany) for anticoagulation at a ratio to blood of 0.14:1. After centrifugation at 3000×*g* for 11.5 min, the plasma component was collected and rapidly frozen as aliquots and stored at −80 °C until use (<6 months). Immediately prior to use, the FFP was thawed and warmed to 37 °C.

### Measurement of TNF-α

Plasma concentration of tumour necrosis factor (TNF)-α was determined by electrochemiluminescence according to the instructions provided by the manufacturer (Mesoscale, Rockville, MD, USA).

### Measurement of plasma volume, blood volume and transcapillary escape rate

Blood samples were collected 2 h after termination of resuscitation and measurement of the plasma volume (PV) and transcapillary escape rate (TER) was performed as described previously [17]. Briefly, the animals received a bolus injection of about 25 kBq (0.05 mg/kg) of human ^125^I-albumin dissolved in 100 μl of 0.9 % NaCl in the internal jugular vein. To determine the exact dose injected, the remaining radioactivity in the emptied vial, the syringe and the needle was subtracted from the total radioactivity in the prepared dose. Arterial blood samples of 250 μl were collected in heparinized vials at 5, 10, 15, 30, 45 and 60 min after the ^125^I-albumin injection. After centrifugation, the radioactivity in 100 μl of plasma was measured in a gamma counter (Wizard 1480, LKB-Wallac, Turku, Finland) and corrections for spill over and background were made automatically. Plasma volume was calculated by dividing the injected dose by the resulting concentration of the tracer at 5 min, and blood volume was calculated as plasma volume/(1-haematocrit). TER was calculated by fitting the decay of ^125^I-albumin at the six sampling points to a linear equation and is expressed in percent per hour. Following TER measurement, the animals were killed by an intravenous injection of 3 M KCl. Six animals showed poor linearity (*r*^2^ < 0.7), and one animal did not survive the complete TER measurement; consequently, the TER measurements for these animals were excluded.

### Measurement of heparan sulphate

A sandwich enzyme immunoassay (Cat. No. 280564–1, AMS Biotechnology, UK) using two monoclonal antibodies specific to heparan sulphate was performed according to the manufacturer’s instructions. Before being pipetted onto the plate, proteins in the plasma samples (20 μl) were digested by adding 2 μl 20 mg/ml Actinase E solution (Sigma-Aldrich, Sweden) in 0.5 M Tris-HCl, pH 7.4, followed by incubation for 20 h at 55 °C. Digestion was stopped by boiling for 5 min. The mixture was centrifuged at 3000×*g*, the supernatant was diluted 1:8 in provided sample diluent and 20 μl was used for the assay. No cross reactivity for heparin was detected, and the coefficient of variation was 6 % in six control replicates. The lower limit of detection was 0.25 μg/ml. Total quantity of circulating heparan sulphate at the end of the experiment was calculated as plasma concentration times the plasma volume.

### Syndecan-1 ELISA

A sandwich enzyme immunoassay (Cat. no. E91966Ra, USCNK, Nordic Diagnostica, Sweden) using an antibody specific to rat syndecan-1 ectodomain was performed according to the manufacturer’s instructions on samples analysed in duplicate and diluted 1:10 in 20 mM PBS, pH 7.1. The manufacturer reports no significant cross reactivity between syndecan-1 and analogues. The lower limit of detection was 1.6 ng/ml, and the coefficient of variation in eight control replicates was 14 %. Total quantity of circulating syndecan-1 at the end of the experiment was calculated as plasma concentration times the plasma volume.

### Statistical analysis

No power analysis was performed prior to the experiments. Number of animals in each group was based on a previous study demonstrating differences in plasma concentration of syndecan-1 following resuscitation with different fluids in a haemorrhage model [8]. Physiological parameters, heparan sulphate and syndecan-1 values, plasma volume and TER had similar means and medians; quartiles were symmetric to the means and passed Lilliefors test for normality and the data were therefore considered to follow the Gaussian distribution. No significant difference in variance was detected between groups as assessed with the Brown-Forsythe test. To evaluate changes in physiological parameters, heparan sulphate and syndecan-1 from baseline to after haemorrhage (i.e. prior to randomization), data from all animals were pooled and analysed using a paired Student’s *t* test. To evaluate changes within group changes after haemorrhage, paired Student’s *t* test was used. The analysis was corrected for multiple comparisons using the Bonferroni method. Differences between groups after resuscitation were evaluated with one-way ANOVA followed by post hoc testing using the Newman-Keuls correction. Calculations were performed using GraphPad Prism version 6 (GraphPad Software, San Diego, USA), and all analyses were performed by an investigator blinded to the treatment allocation. *P* values <0.05 were considered statistically significant. Data are expressed as mean ± S.D.

## Results

### Animal model mortality

**Fig. 2:**
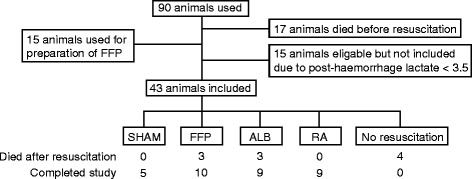
CONSORT flow diagram. *ALB* albumin, *FFP* fresh frozen plasma, *RA* Ringer’s acetate

The mortality following haemorrhage but prior to resuscitation was 24 %. No statistically significant difference in mortality between any of the groups from the start of treatment to the end of experiment was detected (*P* = 0.27, chi-square, Fig. 2).

### Physiological parameters

Physiological data for animals that completed the study are presented in Table 1. Mean arterial pressures, blood gases, haematocrit and lactate were similar in the groups at baseline. At 2.5 h after haemorrhage, mean arterial pressure, central venous oxygen saturation, haematocrit and base excess had decreased while lactate had increased (*P* < 0.05, paired *t* test for all animals). After resuscitation, the mean arterial pressure was higher in colloid resuscitated groups compared to the RA group (Table 1). Lactate concentrations decreased in all groups following resuscitation (*P* < 0.05, paired *t* test) and were lower in the albumin group than in the RA group (Table 1). Central venous oxygen saturation increased following resuscitation in all groups and tended to be higher in the colloid resuscitated groups (*P* = 0.07, ANOVA). Base excess increased in all groups after resuscitation (*P* < 0.05, paired *t* test) and was higher in the FFP group compared to the other groups (Table 1). Haematocrit decreased further after resuscitation in all groups (*P* < 0.05, paired *t* test) and was similar among resuscitated groups. The concentration of TNF-α after haemorrhage (185 ± 152 ng/ml, n = 26) was higher than sham animals (9 ± 9 ng/ml, *n* = 4, *P* < 0.05).

**Table 1:** Physiological parameters

	Baseline	After haemorrhage	Resuscitated	
MAP (mmHg)				
FFP	82 ± 18	53 ± 9	68 ± 11	*^#^
ALB	74 ± 20	54 ± 12	70 ± 10	*^#^
RA 4.5	100 ± 9	50 ± 6	57 ± 7	
SHAM	84 ± 7	100 ± 20	90 ± 18	
ScvO2 (%)				
FFP	82 ± 5	40 ± 9	75 ± 7	*
ALB	78 ± 11	44 ± 14	74 ± 9	*
RA 4.5	85 ± 3	46 ± 9	65 ± 11	*
SHAM	82 ± 7	75 ± 5	76 ± 8	
Lactate (mmol/L)				
FFP	2.2 ± 0.5	6.0 ± 1.9	1.8 ± 0.7	*
ALB	2.2 ± 0.5	6.5 ± 3.3	1.2 ± 0.5	*^#^
RA 4.5	2.0 ± 0.7	5.4 ± 1.1	2.4 ± 1.4	*
SHAM	1.8 ± 0.3	2.1 ± 1.1	1.8 ± 0.2	
Base excess (mmol/L)				
FFP	5.1 ± 1.2	−3.9 ± 3.8	5.9 ± 1.0	*^†^
ALB	5.1 ± 1.4	−4.4 ± 6.4	0.9 ± 1.8	
RA 4.5	5.4 ± 1.4	−2.8 ± 2.6	0.3 ± 2.2	
SHAM	6.0 ± 1.	3.2 ± 1.6	2.2 ± 2.2	
Haematocrit (%)				
FFP	42 ± 1	33 ± 3	19 ± 2	*
ALB	42 ± 3	33 ± 5	18 ± 3	*
RA 4.5	42 ± 1	31 ± 5	20 ± 3	*
SHAM	42 ± 1	39 ± 4	40 ± 6	

Data presented as mean ± SD. The change in values after resuscitation compared to after haemorrhage within groups was evaluated with paired *t* test corrected for multiple analyses with the Bonferroni correction (*P < 0.05). Differences in between groups after resuscitation was analysed with one-way repeated measurement ANOVA followed by post hoc testing using the Newman-Keuls correction

*MAP* mean arterial pressure, *ScvO*_*2*_ central venous oxygen saturation, *FFP* fresh frozen plasma, *ALB* albumin, *RA* Ringer’s acetate

^#^Significant difference compared to the RA group

^†^Significant difference compared to ALB and RA groups, *P* < 0.05

### Plasma and blood volume

**Fig. 3:**
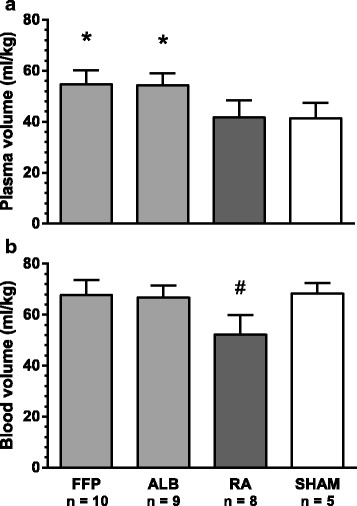
Plasma (**a**) and blood (**b**) volumes in resuscitated and sham animals at the end of the experiment. Data were analysed with one-way ANOVA followed by post hoc testing using the Newman-Keuls correction. Values are means ± SD. *FFP* fresh frozen plasma, *ALB* albumin, *RA* Ringer’s acetate, *significant difference compared to RA and SHAM, ^#^significant difference compared to FFP, ALB and SHAM, *P* < 0.05

Following resuscitation, the plasma volumes in animals resuscitated with albumin and FFP were 55 ± 5 ml/kg and 54 ± 4 ml/kg, respectively, and were higher than the corresponding values in the RA group of 42 ± 7 ml/kg. Plasma volume in the sham animals was 41 ± 6 ml/kg (Fig. 3a). Calculated blood volumes were normalized in animals resuscitated with FFP or albumin (68 ± 6 ml/kg and 67 ± 5 ml/kg, respectively) in relation to sham animals (68 ± 4 ml/kg) while the blood volume was significantly lower in the RA group (52 ± 8 ml/kg, Fig. 3b).

### Circulating levels of heparan sulphate and syndecan-1

Plasma concentrations of heparan sulphate increased from 16 ± 14 μg/ml at baseline to 83 ± 39 μg/ml after haemorrhage (*P* < 0.001, paired *t* test for all animals, Table 2). After resuscitation, plasma concentration of heparan sulphate was higher in animals resuscitated with RA than in animals resuscitated with albumin or FFP (Table 2). After adjusting for differences in plasma volume, however, the total circulating amount of heparan sulphate did not differ between the different resuscitated groups (*P* = 0.4, ANOVA, Table 2). Mean difference in circulating amount of heparan sulphate between the animals resuscitated with FFP and RA and between animals resuscitated with albumin and RA were 853 (95 % CI, −153 to 1858) μg/kg and 449 (95 % CI, −1967 to 1070) μg/kg, respectively.

**Table 2:** Heparan sulphate and syndecan-1 concentrations and total circulating quantity at the end of the experiment

		Concentration			Quantity	
	Baseline	After haemorrhage	Resuscitated		Resuscitated	
HS (μg/ml)					HS (μg/kg)	
FFP	22 ± 20	85 ± 37	53 ± 21	*^#^	2879 ± 1075	^†^
ALB	11 ± 6	88 ± 46	59 ± 33	*^#^	3318 ± 1814	^†^
RA	15 ± 10	79 ± 37	92 ± 28	^†^	3731 ± 777	^†^
SHAM	20 ± 13	37 ± 19	37 ± 25		1683 ± 1396	
Syndecan-1 (ng/ml)					Syndecan-1 (ng/kg)	
FFP	15.3 ± 3.1	20.6 ± 6.1	19.5 ± 4.2		1061 ± 219	^†^
ALB	15.1 ± 4.8	16.5 ± 4.0	18.0 ± 5.0		970 ± 240	^†^
RA	16.1 ± 3.3	17.5 ± 4.5	22.6 ± 6.3	*^†^	906 ± 253	^†^
SHAM	16.3 ± 2.0	16.3 ± 3.1	14.5 ± 1.9		605 ± 145	

Values are means ± SD. The change in concentration after resuscitation compared to after haemorrhage within groups was evaluated with paired *t* test corrected for multiple analyses with the Bonferroni correction (*P < 0.05). Differences in between groups after resuscitation was analysed with one-way repeated measurement ANOVA followed by post hoc testing using the Newman-Keuls correction

*FFP* fresh frozen plasma, *ALB* albumin, *RA* Ringer’s acetate

^#^Significant difference compared to the Ringer’s acetate group

^†^Significant difference compared to SHAM, *P* < 0.05

**Fig. 4:**
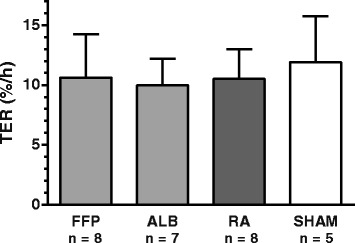
Transcapillary escape rate (TER) for ^125^I-labelled human serum albumin at the end of the experiment. No difference between groups was detected with one-way repeated measurement ANOVA. Values are means ± SD. *FFP* fresh frozen plasma, *ALB* albumin, *RA* Ringer’s acetate

Syndecan-1 levels were increased after haemorrhage compared to baseline and were 18.3 ± 5.2 ng/ml and 15.5 ± 3.6 ng/ml, respectively (*P* = 0.006, paired *t* test). No differences in syndecan-1 concentrations could be detected between the different groups after resuscitation (*P* = 0.1, ANOVA, Fig. 4b). Total amount of circulating syndecan-1 did not differ between the different resuscitated groups (*P* = 0.4, ANOVA, Table 2). Mean difference in amount of circulating syndecan-1 between the animals resuscitated with FFP and RA and between animals resuscitated with albumin and RA were 154 (95 % CI, −74 to 383) ng/kg and 64 (95 % CI, −192 to 320) ng/kg, respectively.

### Transcapillary escape rate

In the resuscitated animals, the TER was 10.6 ± 3.6 %/h for FFP animals, 10.0 ± 2.2 % for albumin animals and 10.5 ± 2.5 % for RA animals. There were no differences between resuscitated groups or compared to sham animals (11.9 ± 3.9 %/h) (Fig. 4).

## Discussion

The present study showed that the plasma concentrations of markers of endothelial damage were increased in a model of severe haemorrhagic shock. Plasma concentrations of heparan sulphate were higher in animals resuscitated with Ringer’s acetate compared to FFP or albumin. The total amount of circulating heparan sulphate did not, however, differ between the resuscitated groups. Resuscitation with FFP or albumin in a ratio of 1.25:1 and 1:1, respectively, to the blood loss resulted in equal plasma volume expansion whereas resuscitation with Ringer’s acetate in a ratio of 4.5:1 to the blood loss resulted in less plasma volume expansion than in the colloid groups. No differences in TER for albumin between the resuscitated animals could be detected.

The volume of haemorrhage was chosen to mimic the clinical scenario with a life-threatening haemorrhage in which early transfusion of blood products have been suggested to be beneficial [2]. Based on the high mortality both prior to resuscitation and after resuscitation, the high lactate values and low central venous oxygen saturation prior to resuscitation, we conclude that the model fulfilled our objective. The observation that blood pressure was not normalized following resuscitation to a normal blood volume suggests that the haemorrhage caused a systemic inflammatory reaction with decreased vascular resistance and/or decreased cardiac contractility [18]. This is also supported by our result of an increase in plasma concentration of the pro-inflammatory cytokine, TNF-α after haemorrhage.

The dosing of RA based on a previous study by us and others was intended to result in a similar plasma volume expansion as in the two colloid groups so that potential effects of the colloid infusions on the glycocalyx would not be confounded by differences in plasma volume [15, 19]. The result that plasma volume in the RA group was lower than in the colloid groups was therefore unexpected. Because the endothelium of all tissues, except the CNS, is freely permeable to electrolytes, crystalloids are commonly thought to distribute rapidly in the whole extravascular volume. Hence, the volume of crystalloid remaining intravascular immediately after resuscitation should reflect the ratio of the extravascular to intravascular distribution volumes. This means that an increased extravascular distribution volume of crystalloids secondary to a shock-induced SIRS response could explain the low efficacy of crystalloids as plasma volume expanders and such a change in the interstitium has been hypothesized to be an important contributor to inflammation-induced oedema formation [20]. Support for this mechanism in inflammatory conditions may be found in a recent publication from our group in which we found that at 15 min after resuscitation in severe sepsis, only about 8 % of the infused volume of crystalloids remained intravascular [21].

Since we did not measure plasma volume immediately after resuscitation but waited 2 h in the present study, other more slowly acting mechanisms may have contributed to the poor plasma volume expanding effect of crystalloids compared to colloids. Infusion of crystalloids will decrease the colloid osmotic force that counteracts filtration of fluid and is likely to further decrease plasma volume expansion by crystalloids. Also, we cannot exclude that different effects on permeability by FFP and colloids on the one hand and crystalloids on the other hand could influence plasma volume expansion. If present, such an effect cannot be specific for FFP since albumin is equally effective as volume expander. Also, no support for such an effect can be found in TER data. In summary, we believe that an inflammation-induced increase in the extravascular distribution volume crystalloids in combination with a low plasma colloid osmotic pressure is the most likely reason for the poor plasma volume expanding properties of RA.

The importance of the difference in plasma volume expansion by the colloids and crystalloids may be supported the higher blood pressure and the observed trend towards improvement in parameters reflecting adequacy of cardiac output, i.e. central venous oxygen saturation and clearance of lactate. The potential clinical importance of this observation may be illustrated by several studies showing that high plasma lactate and low central venous oxygen saturation are associated with poor outcome in trauma patients [22–25]. Given that plasma volume was similar in the albumin and FFP groups, we hypothesize that the significant increase in BE after resuscitation with FFP is related to the citrate and/or to the buffering capacity of other components of FFP [26, 27]. As we did not transfuse erythrocytes, restoration of normovolemia can only be accomplished by an increase in plasma volume above baseline. The observation that FFP and ALB animals had similar blood volumes as the sham group suggests that we did not over resuscitate the animals.

Heparan sulphate is a major component of endothelial proteoglycans, and plasma concentration of heparan sulphate may therefore be regarded as a marker of generalized shedding of proteoglycans from the endothelium. To our knowledge, this is the first report of heparan sulphate levels in rat plasma. The observation that baseline levels in rats are about twofold higher compared to reports using human controls patients using the same detection method indicates that species-dependent differences in heparan sulphate metabolism may exist [28–30].

By measuring plasma volume, we could calculate total circulating amount of heparan sulphate, and our result supports the concept that constituents of the glycocalyx are shedded in haemorrhagic shock [8, 31]. Similarly, the increased plasma concentrations of heparan sulphate in crystalloid-treated animals compared to FFP and albumin are in line with previous results [8, 31]. It is, however, possible that the interpretation of plasma concentration is confounded by differences in distribution volume, and we did, therefore, calculate the total circulating quantity of heparan sulphate. Our result of similar amount of circulating heparan sulphate in animals resuscitated with colloids and crystalloids suggests that differences in heparan sulphate concentrations, to a large extent, reflect differences in distribution volume in the different groups.

In an attempt to identify the molecular source of heparan sulphate, we also measured syndecan-1 levels in plasma. The rationale for choosing syndecan-1 was that high levels of syndecan-1 have been associated with poor outcome in trauma patients and that syndecan-1 is expressed on endothelial cells as well as being a major proteoglycan within the cardiovascular system [6, 32, 33]. The observation that plasma syndecan-1 levels did not differ between animals resuscitated with FFP and animals resuscitated crystalloids is in contrast to the results from two recently published studies using a similar experimental design in which it was demonstrated that resuscitation with FFP is accompanied with lower plasma levels of syndecan-1 compared to resuscitation with Ringer’s lactate [8, 31]. Subtle differences in experimental design could explain the differences in results such as differences in timing of plasma sampling.

Our result of no effect on TER by haemorrhage could be taken to indicate that permeability is unchanged in our model despite the severity of the haemorrhage. However, given that the model induces an inflammatory response with increased levels of the cytokine TNF-α as well as increased plasma levels of markers of endothelial dysfunction, we think that this is unlikely. It should be noted that extravasation of albumin occurs both by diffusion and convection of which the latter is influenced by arterial pressure [34], and it is more likely that the lower blood pressure in the haemorrhaged groups may have offset the effect of a permeability increase on TER for albumin. Our previous finding of a trauma-induced increase TER by about 30 % in a model in which mean arterial pressure was maintained may support the plausibility of this hypothesis. This is a change with the same range as the decrease in blood pressure in the haemorrhaged groups compared to the sham group. Importantly, our results of a similar TER in all resuscitated groups indicate that the superior plasma volume expansion by colloids is not due to reduced vascular leakage of albumin.

### Limitations

It should be recognized that our study suffers from several limitations. We only measured markers of glycocalyx degradation at one time point after resuscitation, and it is possible that beneficial effects of FFP on markers of glycocalyx degradation could have been detected if observation time had been longer or shorter. Also, although several studies have documented degradation of endothelial glycocalyx following haemorrhage by electron microscopy, we cannot be sure that the endothelium is the major source of heparan sulphate or syndecan-1 [35].

Assuming that shedding of the glycocalyx is positively correlated with severity of shock, we acknowledge the possibility that mortality after resuscitation may have introduced a bias and decreased levels of syndecan-1 and heparan sulphate. However, because mortality after resuscitation was similar in the different groups, such an effect is unlikely to influence the difference in glycocalyx degradation products and is unlikely to influence the validity of our conclusions. Assuming that crystalloid solutions possess glycocalyx releasing properties per se, and given the fact that crystalloids were less efficacious than the colloids to restore blood pressure, we cannot exclude that the amount of heparan sulphate and syndecan-1 would have been higher in the RA group if resuscitation had been targeted to obtain similar blood pressures in all groups.

The relatively wide 95 % confidence intervals of difference in amount of circulating heparan sulphate and syndecan-1 between animals resuscitated with RA and FFP, respectively, suggest we cannot exclude that smaller differences may exist and could have been detected if more animals had been included. Patients with haemorrhagic shock present with significantly larger increases in syndecan-1 concentration compared to controls than observed in the present study [6, 7]. This suggests that species differences in glycocalyx biology may limit the translatability of our results. Also, inhalational anaesthesia has been suggested to mitigate heparan sulphate and syndecan-1 shedding in ischemia reperfusion, and it is therefore possible that our anaesthesia method may decrease the release of heparan sulphate and syndecan-1 [36].

FFP contains endogenous heparan sulphate as well as syndecan-1, and it could be argued that the addition of exogenous heparan sulphate and syndecan-1 could have influenced our results. Assuming that the donor animals had similar plasma concentrations of heparan sulphate and syndecan-1 as the sham animals and that no degradation occurred during the preparation of FFP, it can be calculated that resuscitation added about 420 μg/kg of heparan sulphate and 408 ng/kg of syndecan-1. The transfusion of heparan sulphate would not influence our conclusions even if no exogenous heparan sulphate was cleared from plasma. The exogenous syndecan-1 could potentially influence our result if nothing or very little was cleared from circulation. While there are no data on clearance of syndecan-1 from rat plasma, other proteoglycans appear to be cleared very rapidly from plasma with a half-life of about 1 min after intravenous injection [37]. In humans, it has been shown that syndecan-1 and HS are rapidly cleared from plasma with plasma half-life in the order of less than 15 min [30]. Based on this, we conclude that the transfused syndecan-1 is very unlikely to have influenced our results.

## Conclusions

The results indicate that improved outcome in haemorrhagic shock by FFP in part could be explained by better plasma volume expansion compared to crystalloids. The decrease in plasma concentration of markers of glycocalyx degradation after resuscitation with FFP is largely secondary to differences in plasma volume and may not accurately reflect effects of FFP on the glycocalyx.

## Abbreviations

ALB: albumin
FFP: fresh frozen plasma
MAP: mean arterial pressure
PV: plasma volume
RA: Ringer’s acetate
TER: transcapillary escape rate
